# Early prediction of acute kidney injury after transcatheter aortic valve implantation with urinary G1 cell cycle arrest biomarkers

**DOI:** 10.1186/cc14370

**Published:** 2015-03-16

**Authors:** F Dusse, M Dudásová, D Wendt, M Thielmann, E Demircioglu, HG Jakob, K Pilarczyk

**Affiliations:** 1 University Hospital Essen, Germany

## Introduction

Acute kidney injury (AKI) is a common complication following transcatheter aortic valve implantation (TAVI) and has been shown to increase mortality. The concentration of the G1 cell cycle arrest proteins TIMP-2 and IGFBP7 in the urine have recently been suggested as sensitive biomarkers for early detection of AKI in critically ill patients. Whether postoperatively elevated levels of urinary [TIMP-2][IGFBP7] (UTI) predict the development of an AKI in patients undergoing TAVI is currently unknown.

## Methods

In a prospective cohort study, 40 patients undergoing TAVI, either trans-apical (TA) or trans-aortic (TAo), were enrolled. Serial measurements of UTI were performed every 12 hours in the postoperative course. Results were calculated for their multiplication and presented as arbitrary values. Urinary output and serum creatinine were recorded simultaneously. The primary clinical endpoint was the occurrence of AKI according to the AKI Network.

## Results

Mean age was 81 ± 5.6 years (16 male, 40.0%). Thirty-five patients underwent TA-TAVI and five patients TAo-TAVI. AKI developed in 17 patients (42.5%); seven patients (17.5%) suffered from AKI 3 and required renal replacement therapy (RRT). Mean maximum value of UTI within 24 hours after TAVI was significantly higher in patients with AKI compared with patients without renal impairment (2.19 ± 3.11 vs. 0.67 ± 0.816, *P *= 0.037) and higher in patients with AKI 3 compared with patients with AKI 2 (4.73 ± 3.58 vs. 0.59 ± 0.71, *P *= 0.022). In contrast, preoperative creatinine (AKI (mg/dl) 1.22 ± 0.41 vs. no AKI 1.30 ± 0.59; AKI 3 1.32 ± 0.49 vs. AKI 2 1.26 ± 0.53, *P *= NS) and early postoperative serum creatinine levels (maximum within 24 hours after TAVI: AKI 1.41 ± 0.50 vs. no AKI 1.34 ± 0.60; AKI 3 1.69 ± 0.56 vs. AKI 2 1.32 ± 0.54, *P *= NS) did not show any association with the development of AKI. ROC analyses revealed a very good predictive value of early UTI levels for the development of AKI 3 within the next 72 hours after TAVI with a sensitivity of 100% and a specificity of 80% for a cutoff value of 0.815 (AUC = 0.919, 95% CI = 0.824 to 1.0, SE 0.048, *P *= 0.001).

## Conclusion

Early elevation of urinary [TIMP-2][IGFBP7] after TAVI is associated with the development of postoperative AKI. These biomarkers have an excellent diagnostic accuracy in the prediction of severe AKI requiring RRT that is superior to that of serum creatinine. Further studies are necessary to prove whether UTI-guided therapy of patients with AKI can reduce morbidity and mortality.

